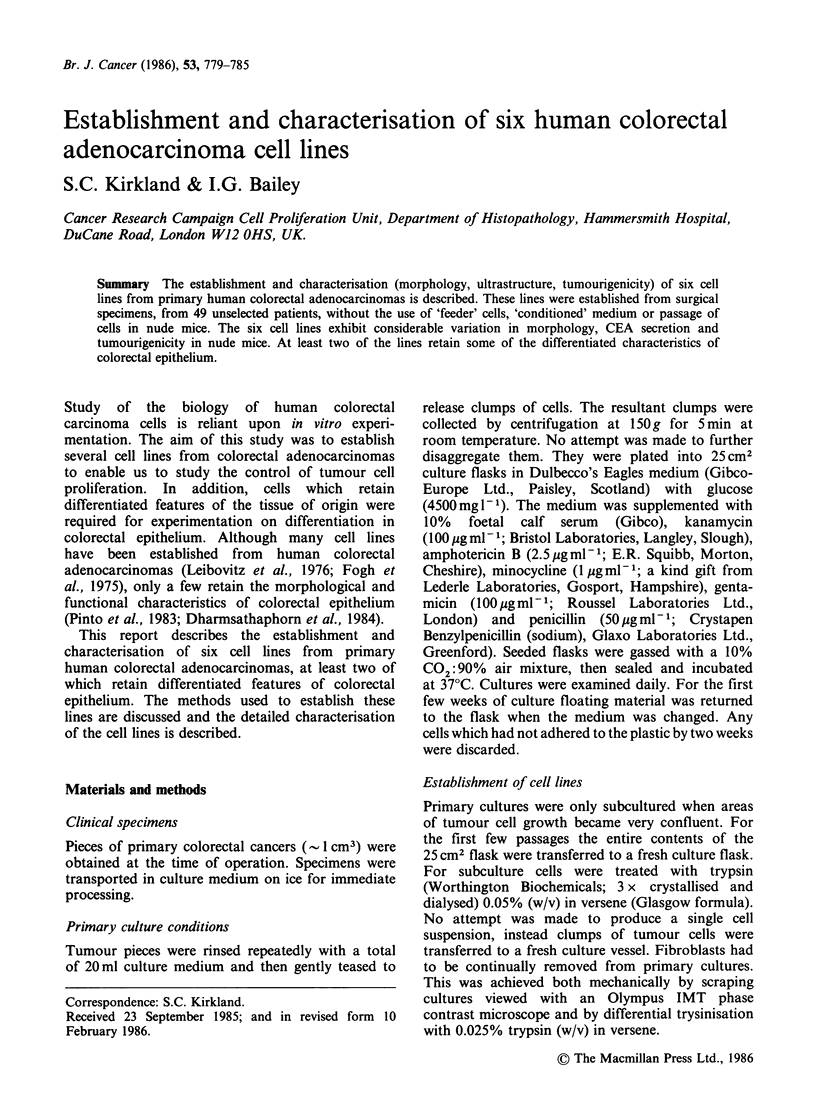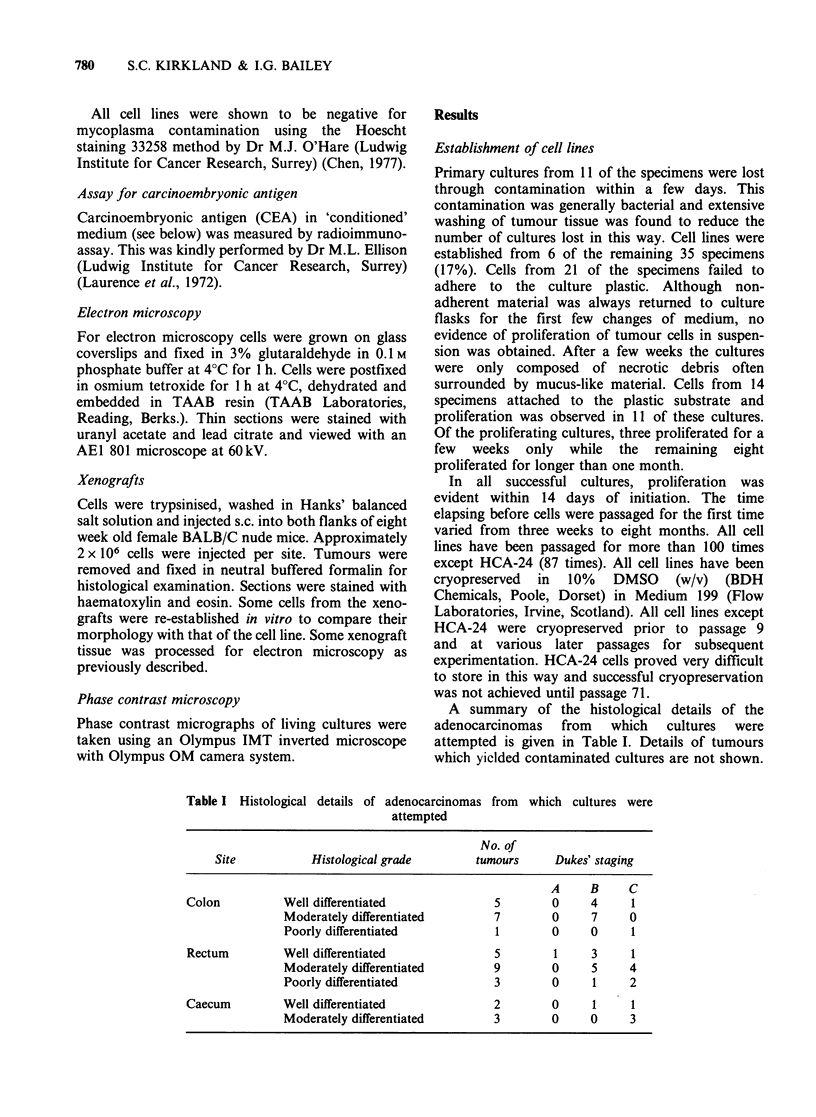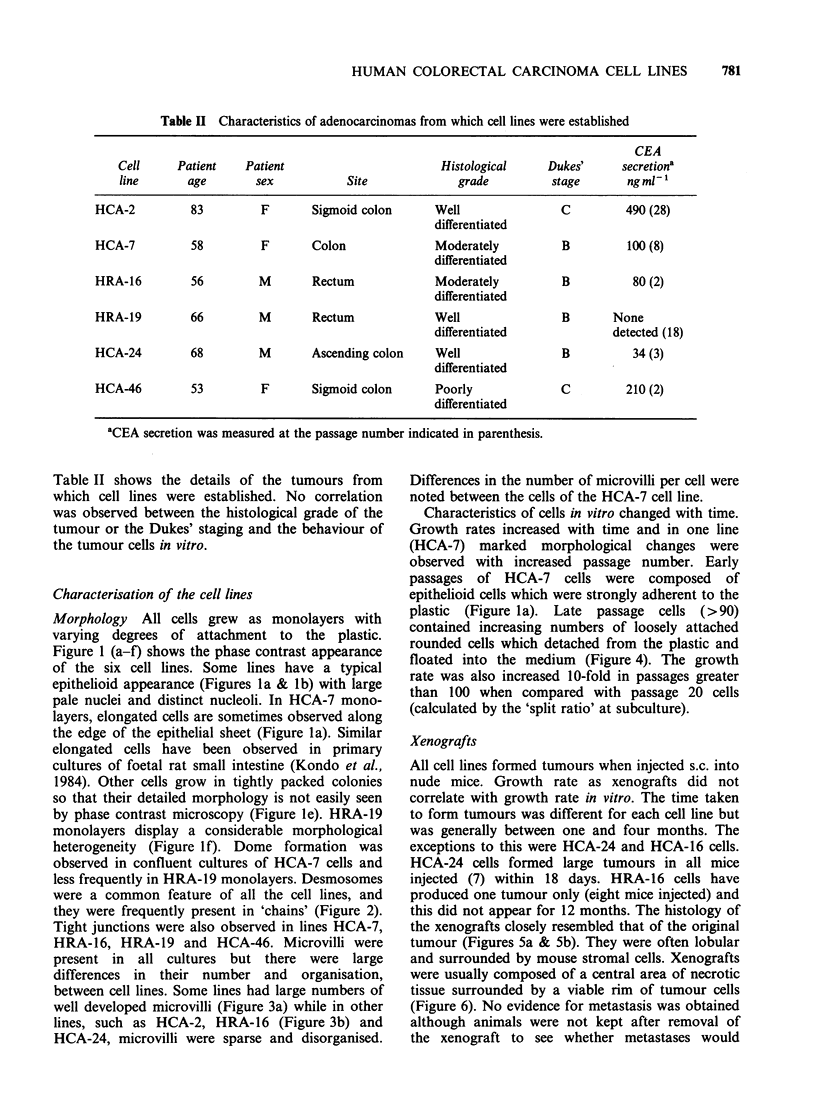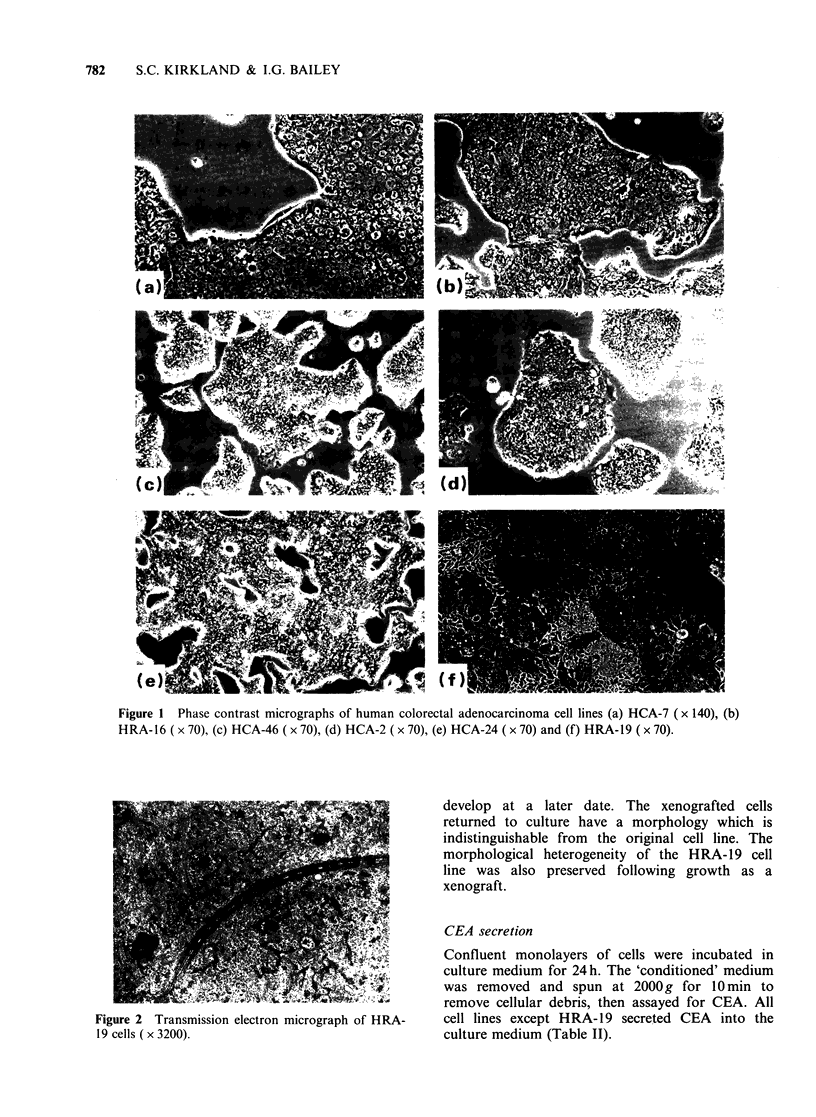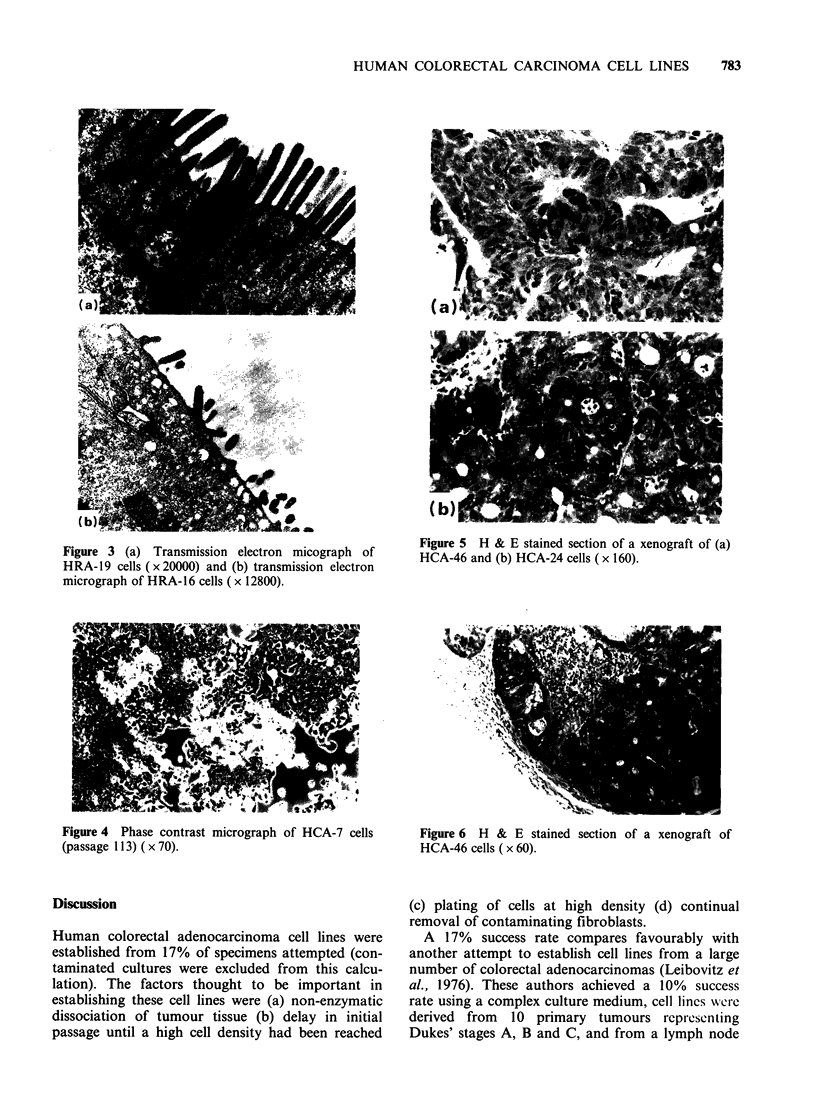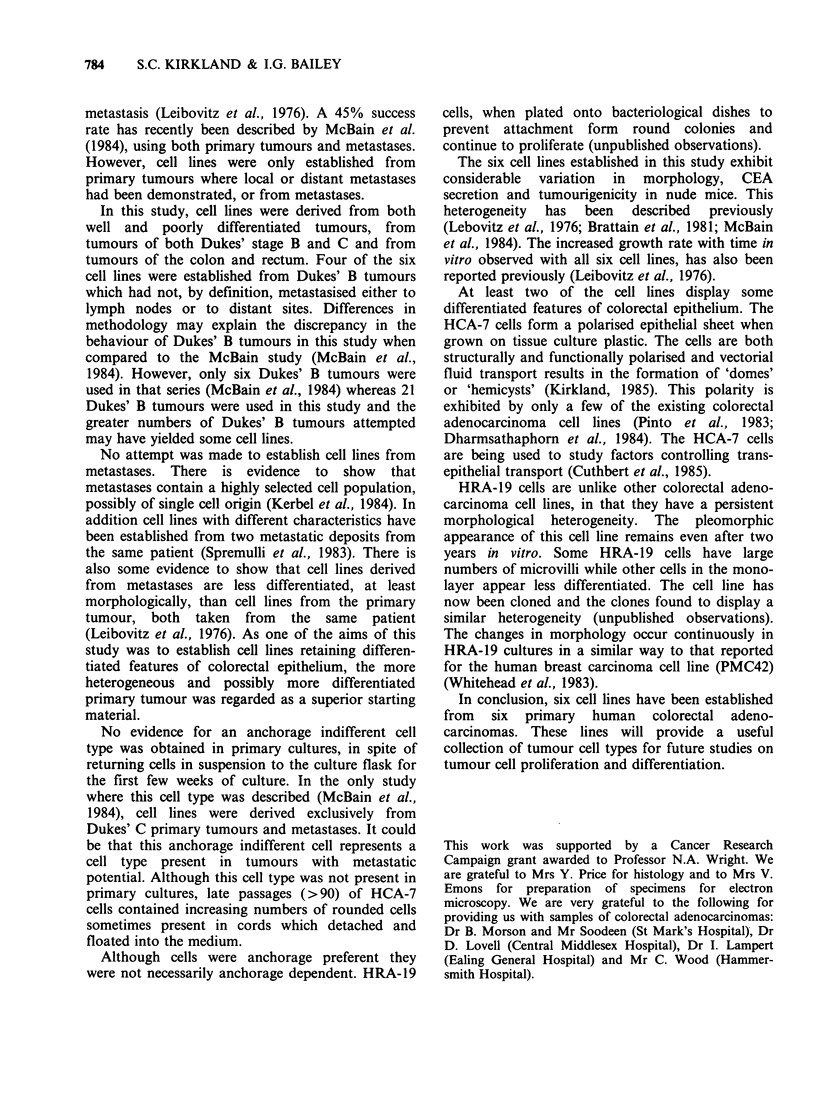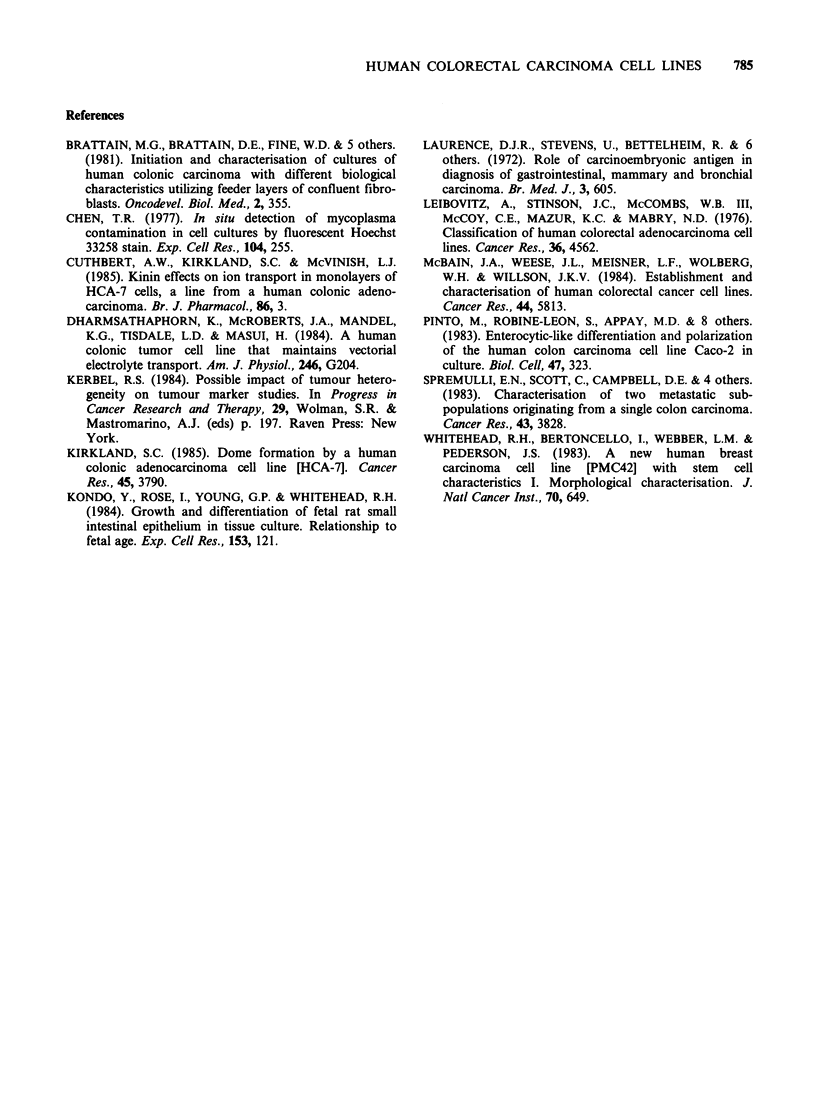# Establishment and characterisation of six human colorectal adenocarcinoma cell lines.

**DOI:** 10.1038/bjc.1986.132

**Published:** 1986-06

**Authors:** S. C. Kirkland, I. G. Bailey

## Abstract

**Images:**


*Br. J. Cancer* (1986), **53**, 779–785

### Establishment and characterisation of six human colorectal adenocarcinoma cell lines

S.C. Kirkland & I.G. Bailey

*Cancer Research Campaign Cell Proliferation Unit, Department of Histopathology, Hammersmith Hospital, DuCane Road, London W12 0HS, UK.*

**Summary** The establishment and characterisation (morphology, ultrastructure, tumourigenicity) of six cell lines from primary human colorectal adenocarcinomas is described. These lines were established from surgical specimens, from 49 unselected patients, without the use of 'feeder' cells, 'conditioned' medium or passage of cells in nude mice. The six cell lines exhibit considerable variation in morphology, CEA secretion and tumourigenicity in nude mice. At least two of the lines retain some of the differentiated characteristics of colorectal epithelium.

Study of the biology of human colorectal carcinoma cells is reliant upon *in vitro* experimentation. The aim of this study was to establish several cell lines from colorectal adenocarcinomas to enable us to study the control of tumour cell proliferation. In addition, cells which retain differentiated features of the tissue of origin were required for experimentation on differentiation in colorectal epithelium. Although many cell lines have been established from human colorectal adenocarcinomas (Leibovitz *et al.*, 1976; Fogh *et al.*, 1975), only a few retain the morphological and functional characteristics of colorectal epithelium (Pinto *et al.*, 1983; Dharmsathaphorn *et al.*, 1984).

This report describes the establishment and characterisation of six cell lines from primary human colorectal adenocarcinomas, at least two of which retain differentiated features of colorectal epithelium. The methods used to establish these lines are discussed and the detailed characterisation of the cell lines is described.

### Materials and methods

### *Clinical specimens*

Pieces of primary colorectal cancers ($\sim 1\,cm^3$) were obtained at the time of operation. Specimens were transported in culture medium on ice for immediate processing.

### *Primary culture conditions*

Tumour pieces were rinsed repeatedly with a total of 20 ml culture medium and then gently teased to release clumps of cells. The resultant clumps were collected by centrifugation at 150 *g* for 5 min at room temperature. No attempt was made to further disaggregate them. They were plated into $25\,cm^2$ culture flasks in Dulbecco's Eagles medium (Gibco-Europe Ltd., Paisley, Scotland) with glucose ($4500\,mg\,l^{-1}$). The medium was supplemented with 10% foetal calf serum (Gibco), kanamycin ($100\,\mu g\,ml^{-1}$; Bristol Laboratories, Langley, Slough), amphotericin B ($2.5\,\mu g\,ml^{-1}$; E.R. Squibb, Morton, Cheshire), minocycline ($1\,\mu g\,ml^{-1}$; a kind gift from Lederle Laboratories, Gosport, Hampshire), gentamicin ($100\,\mu g\,ml^{-1}$; Roussel Laboratories Ltd., London) and penicillin ($50\,\mu g\,ml^{-1}$; Crystapen Benzylpenicillin (sodium), Glaxo Laboratories Ltd., Greenford). Seeded flasks were gassed with a 10% $CO_2$:90% air mixture, then sealed and incubated at 37°C. Cultures were examined daily. For the first few weeks of culture floating material was returned to the flask when the medium was changed. Any cells which had not adhered to the plastic by two weeks were discarded.

### *Establishment of cell lines*

Primary cultures were only subcultured when areas of tumour cell growth became very confluent. For the first few passages the entire contents of the $25\,cm^2$ flask were transferred to a fresh culture flask. For subculture cells were treated with trypsin (Worthington Biochemicals; 3× crystallised and dialysed) 0.05% (w/v) in versene (Glasgow formula). No attempt was made to produce a single cell suspension, instead clumps of tumour cells were transferred to a fresh culture vessel. Fibroblasts had to be continually removed from primary cultures. This was achieved both mechanically by scraping cultures viewed with an Olympus IMT phase contrast microscope and by differential trysinisation with 0.025% trypsin (w/v) in versene.

Correspondence: S.C. Kirkland.
Received 23 September 1985; and in revised form 10 February 1986.

© The Macmillan Press Ltd., 1986

All cell lines were shown to be negative for mycoplasma contamination using the Hoescht staining 33258 method by Dr M.J. O'Hare (Ludwig Institute for Cancer Research, Surrey) (Chen, 1977).

*Assay for carcinoembryonic antigen*

Carcinoembryonic antigen (CEA) in 'conditioned' medium (see below) was measured by radioimmunoassay. This was kindly performed by Dr M.L. Ellison (Ludwig Institute for Cancer Research, Surrey) (Laurence *et al.*, 1972).

*Electron microscopy*

For electron microscopy cells were grown on glass coverslips and fixed in 3% glutaraldehyde in 0.1 M phosphate buffer at 4°C for 1 h. Cells were postfixed in osmium tetroxide for 1 h at 4°C, dehydrated and embedded in TAAB resin (TAAB Laboratories, Reading, Berks.). Thin sections were stained with uranyl acetate and lead citrate and viewed with an AE1 801 microscope at 60 kV.

*Xenografts*

Cells were trypsinised, washed in Hanks' balanced salt solution and injected s.c. into both flanks of eight week old female BALB/C nude mice. Approximately $2 \times 10^6$ cells were injected per site. Tumours were removed and fixed in neutral buffered formalin for histological examination. Sections were stained with haematoxylin and eosin. Some cells from the xenografts were re-established *in vitro* to compare their morphology with that of the cell line. Some xenograft tissue was processed for electron microscopy as previously described.

*Phase contrast microscopy*

Phase contrast micrographs of living cultures were taken using an Olympus IMT inverted microscope with Olympus OM camera system.

### Results

*Establishment of cell lines*

Primary cultures from 11 of the specimens were lost through contamination within a few days. This contamination was generally bacterial and extensive washing of tumour tissue was found to reduce the number of cultures lost in this way. Cell lines were established from 6 of the remaining 35 specimens (17%). Cells from 21 of the specimens failed to adhere to the culture plastic. Although non-adherent material was always returned to culture flasks for the first few changes of medium, no evidence of proliferation of tumour cells in suspension was obtained. After a few weeks the cultures were only composed of necrotic debris often surrounded by mucus-like material. Cells from 14 specimens attached to the plastic substrate and proliferation was observed in 11 of these cultures. Of the proliferating cultures, three proliferated for a few weeks only while the remaining eight proliferated for longer than one month.

In all successful cultures, proliferation was evident within 14 days of initiation. The time elapsing before cells were passaged for the first time varied from three weeks to eight months. All cell lines have been passaged for more than 100 times except HCA-24 (87 times). All cell lines have been cryopreserved in 10% DMSO (w/v) (BDH Chemicals, Poole, Dorset) in Medium 199 (Flow Laboratories, Irvine, Scotland). All cell lines except HCA-24 were cryopreserved prior to passage 9 and at various later passages for subsequent experimentation. HCA-24 cells proved very difficult to store in this way and successful cryopreservation was not achieved until passage 71.

A summary of the histological details of the adenocarcinomas from which cultures were attempted is given in Table I. Details of tumours which yielded contaminated cultures are not shown.

**Table I** Histological details of adenocarcinomas from which cultures were attempted

| *Site* | *Histological grade* | *No. of tumours* | *Dukes' staging* | | |
|---|---|---|---|---|---|
| | | | *A* | *B* | *C* |
| Colon | Well differentiated | 5 | 0 | 4 | 1 |
| | Moderately differentiated | 7 | 0 | 7 | 0 |
| | Poorly differentiated | 1 | 0 | 0 | 1 |
| Rectum | Well differentiated | 5 | 1 | 3 | 1 |
| | Moderately differentiated | 9 | 0 | 5 | 4 |
| | Poorly differentiated | 3 | 0 | 1 | 2 |
| Caecum | Well differentiated | 2 | 0 | 1 | 1 |
| | Moderately differentiated | 3 | 0 | 0 | 3 |

**Table II** Characteristics of adenocarcinomas from which cell lines were established

| *Cell line* | *Patient age* | *Patient sex* | *Site* | *Histological grade* | *Dukes' stage* | *CEA secretion*[a] *ng ml*$^{-1}$ |
|---|---|---|---|---|---|---|
| HCA-2 | 83 | F | Sigmoid colon | Well differentiated | C | 490 (28) |
| HCA-7 | 58 | F | Colon | Moderately differentiated | B | 100 (8) |
| HRA-16 | 56 | M | Rectum | Moderately differentiated | B | 80 (2) |
| HRA-19 | 66 | M | Rectum | Well differentiated | B | None detected (18) |
| HCA-24 | 68 | M | Ascending colon | Well differentiated | B | 34 (3) |
| HCA-46 | 53 | F | Sigmoid colon | Poorly differentiated | C | 210 (2) |

[a]CEA secretion was measured at the passage number indicated in parenthesis.

Table II shows the details of the tumours from which cell lines were established. No correlation was observed between the histological grade of the tumour or the Dukes' staging and the behaviour of the tumour cells *in vitro*.

### *Characterisation of the cell lines*

*Morphology* All cells grew as monolayers with varying degrees of attachment to the plastic. Figure 1 (a–f) shows the phase contrast appearance of the six cell lines. Some lines have a typical epithelioid appearance (Figures 1a & 1b) with large pale nuclei and distinct nucleoli. In HCA-7 monolayers, elongated cells are sometimes observed along the edge of the epithelial sheet (Figure 1a). Similar elongated cells have been observed in primary cultures of foetal rat small intestine (Kondo *et al.*, 1984). Other cells grow in tightly packed colonies so that their detailed morphology is not easily seen by phase contrast microscopy (Figure 1e). HRA-19 monolayers display a considerable morphological heterogeneity (Figure 1f). Dome formation was observed in confluent cultures of HCA-7 cells and less frequently in HRA-19 monolayers. Desmosomes were a common feature of all the cell lines, and they were frequently present in 'chains' (Figure 2). Tight junctions were also observed in lines HCA-7, HRA-16, HRA-19 and HCA-46. Microvilli were present in all cultures but there were large differences in their number and organisation, between cell lines. Some lines had large numbers of well developed microvilli (Figure 3a) while in other lines, such as HCA-2, HRA-16 (Figure 3b) and HCA-24, microvilli were sparse and disorganised. Differences in the number of microvilli per cell were noted between the cells of the HCA-7 cell line.

Characteristics of cells *in vitro* changed with time. Growth rates increased with time and in one line (HCA-7) marked morphological changes were observed with increased passage number. Early passages of HCA-7 cells were composed of epithelioid cells which were strongly adherent to the plastic (Figure 1a). Late passage cells (>90) contained increasing numbers of loosely attached rounded cells which detached from the plastic and floated into the medium (Figure 4). The growth rate was also increased 10-fold in passages greater than 100 when compared with passage 20 cells (calculated by the 'split ratio' at subculture).

### *Xenografts*

All cell lines formed tumours when injected s.c. into nude mice. Growth rate as xenografts did not correlate with growth rate *in vitro*. The time taken to form tumours was different for each cell line but was generally between one and four months. The exceptions to this were HCA-24 and HCA-16 cells. HCA-24 cells formed large tumours in all mice injected (7) within 18 days. HRA-16 cells have produced one tumour only (eight mice injected) and this did not appear for 12 months. The histology of the xenografts closely resembled that of the original tumour (Figures 5a & 5b). They were often lobular and surrounded by mouse stromal cells. Xenografts were usually composed of a central area of necrotic tissue surrounded by a viable rim of tumour cells (Figure 6). No evidence for metastasis was obtained although animals were not kept after removal of the xenograft to see whether metastases would

**Figure 1** Phase contrast micrographs of human colorectal adenocarcinoma cell lines (a) HCA-7 ( × 140), (b) HRA-16 ( × 70), (c) HCA-46 ( × 70), (d) HCA-2 ( × 70), (e) HCA-24 ( × 70) and (f) HRA-19 ( × 70).

**Figure 2** Transmission electron micrograph of HRA-19 cells ( × 3200).

develop at a later date. The xenografted cells returned to culture have a morphology which is indistinguishable from the original cell line. The morphological heterogeneity of the HRA-19 cell line was also preserved following growth as a xenograft.

*CEA secretion*

Confluent monolayers of cells were incubated in culture medium for 24 h. The 'conditioned' medium was removed and spun at 2000 *g* for 10 min to remove cellular debris, then assayed for CEA. All cell lines except HRA-19 secreted CEA into the culture medium (Table II).

**Figure 3** (a) Transmission electron micograph of HRA-19 cells ( x 20000) and (b) transmission electron micrograph of HRA-16 cells ( x 12800).

**Figure 5** H & E stained section of a xenograft of (a) HCA-46 and (b) HCA-24 cells ( x 160).

**Figure 4** Phase contrast micrograph of HCA-7 cells (passage 113) ( x 70).

**Figure 6** H & E stained section of a xenograft of HCA-46 cells ( x 60).

### Discussion

Human colorectal adenocarcinoma cell lines were established from 17% of specimens attempted (contaminated cultures were excluded from this calculation). The factors thought to be important in establishing these cell lines were (a) non-enzymatic dissociation of tumour tissue (b) delay in initial passage until a high cell density had been reached (c) plating of cells at high density (d) continual removal of contaminating fibroblasts.

A 17% success rate compares favourably with another attempt to establish cell lines from a large number of colorectal adenocarcinomas (Leibovitz *et al.*, 1976). These authors achieved a 10% success rate using a complex culture medium, cell lines were derived from 10 primary tumours representing Dukes' stages A, B and C, and from a lymph node metastasis (Leibovitz *et al.*, 1976). A 45% success rate has recently been described by McBain *et al.* (1984), using both primary tumours and metastases. However, cell lines were only established from primary tumours where local or distant metastases had been demonstrated, or from metastases.

In this study, cell lines were derived from both well and poorly differentiated tumours, from tumours of both Dukes' stage B and C and from tumours of the colon and rectum. Four of the six cell lines were established from Dukes' B tumours which had not, by definition, metastasised either to lymph nodes or to distant sites. Differences in methodology may explain the discrepancy in the behaviour of Dukes' B tumours in this study when compared to the McBain study (McBain *et al.*, 1984). However, only six Dukes' B tumours were used in that series (McBain *et al.*, 1984) whereas 21 Dukes' B tumours were used in this study and the greater numbers of Dukes' B tumours attempted may have yielded some cell lines.

No attempt was made to establish cell lines from metastases. There is evidence to show that metastases contain a highly selected cell population, possibly of single cell origin (Kerbel *et al.*, 1984). In addition cell lines with different characteristics have been established from two metastatic deposits from the same patient (Spremulli *et al.*, 1983). There is also some evidence to show that cell lines derived from metastases are less differentiated, at least morphologically, than cell lines from the primary tumour, both taken from the same patient (Leibovitz *et al.*, 1976). As one of the aims of this study was to establish cell lines retaining differentiated features of colorectal epithelium, the more heterogeneous and possibly more differentiated primary tumour was regarded as a superior starting material.

No evidence for an anchorage indifferent cell type was obtained in primary cultures, in spite of returning cells in suspension to the culture flask for the first few weeks of culture. In the only study where this cell type was described (McBain *et al.*, 1984), cell lines were derived exclusively from Dukes' C primary tumours and metastases. It could be that this anchorage indifferent cell represents a cell type present in tumours with metastatic potential. Although this cell type was not present in primary cultures, late passages (>90) of HCA-7 cells contained increasing numbers of rounded cells sometimes present in cords which detached and floated into the medium.

Although cells were anchorage preferent they were not necessarily anchorage dependent. HRA-19 cells, when plated onto bacteriological dishes to prevent attachment form round colonies and continue to proliferate (unpublished observations).

The six cell lines established in this study exhibit considerable variation in morphology, CEA secretion and tumourigenicity in nude mice. This heterogeneity has been described previously (Lebovitz *et al.*, 1976; Brattain *et al.*, 1981; McBain *et al.*, 1984). The increased growth rate with time *in vitro* observed with all six cell lines, has also been reported previously (Leibovitz *et al.*, 1976).

At least two of the cell lines display some differentiated features of colorectal epithelium. The HCA-7 cells form a polarised epithelial sheet when grown on tissue culture plastic. The cells are both structurally and functionally polarised and vectorial fluid transport results in the formation of 'domes' or 'hemicysts' (Kirkland, 1985). This polarity is exhibited by only a few of the existing colorectal adenocarcinoma cell lines (Pinto *et al.*, 1983; Dharmsathaphorn *et al.*, 1984). The HCA-7 cells are being used to study factors controlling trans-epithelial transport (Cuthbert *et al.*, 1985).

HRA-19 cells are unlike other colorectal adenocarcinoma cell lines, in that they have a persistent morphological heterogeneity. The pleomorphic appearance of this cell line remains even after two years *in vitro*. Some HRA-19 cells have large numbers of microvilli while other cells in the monolayer appear less differentiated. The cell line has now been cloned and the clones found to display a similar heterogeneity (unpublished observations). The changes in morphology occur continuously in HRA-19 cultures in a similar way to that reported for the human breast carcinoma cell line (PMC42) (Whitehead *et al.*, 1983).

In conclusion, six cell lines have been established from six primary human colorectal adenocarcinomas. These lines will provide a useful collection of tumour cell types for future studies on tumour cell proliferation and differentiation.

This work was supported by a Cancer Research Campaign grant awarded to Professor N.A. Wright. We are grateful to Mrs Y. Price for histology and to Mrs V. Emons for preparation of specimens for electron microscopy. We are very grateful to the following for providing us with samples of colorectal adenocarcinomas: Dr B. Morson and Mr Soodeen (St Mark's Hospital), Dr D. Lovell (Central Middlesex Hospital), Dr I. Lampert (Ealing General Hospital) and Mr C. Wood (Hammersmith Hospital).

**References**

BRATTAIN, M.G., BRATTAIN, D.E., FINE, W.D. & 5 others. (1981). Initiation and characterisation of cultures of human colonic carcinoma with different biological characteristics utilizing feeder layers of confluent fibroblasts. *Oncodevel. Biol. Med.*, **2**, 355.

CHEN, T.R. (1977). *In situ* detection of mycoplasma contamination in cell cultures by fluorescent Hoechst 33258 stain. *Exp. Cell Res.*, **104**, 255.

CUTHBERT, A.W., KIRKLAND, S.C. & McVINISH, L.J. (1985). Kinin effects on ion transport in monolayers of HCA-7 cells, a line from a human colonic adenocarcinoma. *Br. J. Pharmacol.*, **86**, 3.

DHARMSATHAPHORN, K., McROBERTS, J.A., MANDEL, K.G., TISDALE, L.D. & MASUI, H. (1984). A human colonic tumor cell line that maintains vectorial electrolyte transport. *Am. J. Physiol.*, **246**, G204.

KERBEL, R.S. (1984). Possible impact of tumour heterogeneity on tumour marker studies. In *Progress in Cancer Research and Therapy*, **29**, Wolman, S.R. & Mastromarino, A.J. (eds) p. 197. Raven Press: New York.

KIRKLAND, S.C. (1985). Dome formation by a human colonic adenocarcinoma cell line [HCA-7]. *Cancer Res.*, **45**, 3790.

KONDO, Y., ROSE, I., YOUNG, G.P. & WHITEHEAD, R.H. (1984). Growth and differentiation of fetal rat small intestinal epithelium in tissue culture. Relationship to fetal age. *Exp. Cell Res.*, **153**, 121.

LAURENCE, D.J.R., STEVENS, U., BETTELHEIM, R. & 6 others. (1972). Role of carcinoembryonic antigen in diagnosis of gastrointestinal, mammary and bronchial carcinoma. *Br. Med. J.*, **3**, 605.

LEIBOVITZ, A., STINSON, J.C., McCOMBS, W.B. III, McCOY, C.E., MAZUR, K.C. & MABRY, N.D. (1976). Classification of human colorectal adenocarcinoma cell lines. *Cancer Res.*, **36**, 4562.

McBAIN, J.A., WEESE, J.L., MEISNER, L.F., WOLBERG, W.H. & WILLSON, J.K.V. (1984). Establishment and characterisation of human colorectal cancer cell lines. *Cancer Res.*, **44**, 5813.

PINTO, M., ROBINE-LEON, S., APPAY, M.D. & 8 others. (1983). Enterocytic-like differentiation and polarization of the human colon carcinoma cell line Caco-2 in culture. *Biol. Cell*, **47**, 323.

SPREMULLI, E.N., SCOTT, C., CAMPBELL, D.E. & 4 others. (1983). Characterisation of two metastatic subpopulations originating from a single colon carcinoma. *Cancer Res.*, **43**, 3828.

WHITEHEAD, R.H., BERTONCELLO, I., WEBBER, L.M. & PEDERSON, J.S. (1983). A new human breast carcinoma cell line [PMC42] with stem cell characteristics I. Morphological characterisation. *J. Natl Cancer Inst.*, **70**, 649.